# Stabilin-1 in Tumor-Associated Macrophages: A Potential Therapeutic Target in Cancer Immunotherapy

**DOI:** 10.3390/biology14091198

**Published:** 2025-09-05

**Authors:** Jampa Lhamo Gurung, Raju Lama Tamang, Lepakshe Madduri, Robert G. Bennett, Edward N. Harris, Paul W. Denton, Benita McVicker

**Affiliations:** 1 Department of Pathology, Microbiology and Immunology, University of Nebraska Medical Center, Omaha, NE 68198, USA; jgurung@unmc.edu; 2 Department of Internal Medicine, University of Nebraska Medical Center, Omaha, NE 68198, USA; raju.lama@unmc.edu (R.L.T.); s.madduri@unmc.edu (L.M.); rgbennet@unmc.edu (R.G.B.); 3 Research Service, Nebraska-Western Iowa Health Care System, Omaha, NE 68105, USA; 4 Department of Biochemistry and Molecular Biology, University of Nebraska Medical Center, Omaha, NE 68198, USA; 5 Department of Biochemistry, University of Nebraska Lincoln, Lincoln, NE 68588, USA; eharris5@unl.edu; 6 Department of Biology, University of Nebraska Omaha, Omaha, NE 68182, USA; pdenton@unomaha.edu

**Keywords:** stabilin-1, cancer, macrophages, tumor-associated macrophages, immunotherapy, CLEVER-1

## Abstract

Stabilin-1 (STAB1) is a multifunctional scavenger receptor expressed by endothelial cells of the liver, spleen, lymph nodes, bone marrow and a subset of macrophages. STAB1 interacts with different ligands and modulates a wide range of functions including cell trafficking, endocytosis, homeostasis, angiogenesis, and tumor vascularization. The role of STAB1 in cancer progression and metastasis first became evident in *Stab1* knockout (KO) mice, which developed smaller primary tumors and metastatic foci for some cancers. To date, various clinical cohorts and preclinical rodent studies have shown that STAB1 inhibition is associated with elevated anti-tumor T-cell responses. Moreover, human trials using anti-STAB1 antibody treatment indicate a shift towards immune activation and the potential to overcome cancer treatment resistance experienced with other immunotherapies. Although the role of STAB1 in cancer development and metastasis remains to be defined, STAB1 signaling in tumor-associated macrophages and downstream immune modulation are thought to be crucial mechanisms. Herein, we discuss the role of STAB1 in tumor-associated macrophages in relationship to disease progression and patient outcome.

## Simple Summary

Immunotherapy has been a revolutionary approach to combat various cancers. However, not all malignancies respond to immunotherapy, indicating the need for better therapeutic options. Several studies have shown that immune cells within the microenvironment of tumors play an important role in cancer cell survival and response to treatments. It is evident that among the immune cells, tumor-associated macrophages are increasingly recognized for their role in immunotherapy resistance. This review provides a comprehensive overview of tumor-associated macrophages and the role of Stabilin-1-expressing macrophages in tumor development and metastasis. Furthermore, we discuss how targeting Stabilin-1 may represent a potential therapeutic approach for limiting cancer development and advancement.

## 1. Introduction

The immune system acts as a gatekeeper of overall health, maintaining homeostasis by neutralizing foreign antigens and regulating inflammatory responses. When there is dysregulation in the immune system, disease-promoting consequences can occur, including cancer development and progression [1]. The growth and spread of tumor cells are often associated with the evasion or suppression of immune responses [1]. A deeper understanding of the relationship between immune surveillance, immune checkpoints, and tumor progression has led to the emergence of immunotherapy as a powerful strategy for combating cancer. Significant discoveries to date include immune checkpoint inhibitors, chimeric antigen receptor T-cell therapy, and tumor vaccines that have revolutionized cancer treatments [2]. However, not all tumor types respond to immunotherapy, leading to the continued search for alternative strategies [3]. Recent research has shown a heightened interest in understanding the composition and role of immune cells within the complex tumor microenvironment (TME) to understand the underlying mechanisms for tumor survival better and to improve response to therapy [2,3].

The TME is a dynamic system orchestrated by complex components, including stromal cells, CD4+ and CD8+ T cells, natural killer (NK) cells, tumor-related endothelial cells, cancer-associated fibroblasts (CAFs), and tumor-associated macrophages (TAMs) [3,4]. Among the innate immune cells, TAMs are a major component of the TME and key cells involved in cancer immunology [5]. TAMs exhibit extreme adaptability in response to microenvironment stimuli, leading to polarization into particular phenotypes (e.g., the M1-type, which is predominantly pro-inflammatory, and the M2-type, which facilitates anti-inflammatory activities) [6]. In many tumors, the TAM populations are predominantly polarized to alternatively activated M2-type macrophages [5,6]. Evidence has shown that TAMs are involved in immune suppression and are key regulators of tumor growth, angiogenesis, invasion, metastasis, and response to therapy [5]. Therefore, understanding the composition and role of TAMs in the TME is notably important in the search for targetable mechanisms to overcome cancer progression [7].

In macrophage and TAM cell biology, STAB1 is known as fasciclin, epidermal growth factor (EGF)-like, laminin-type EGF-like and Link domain-containing scavenger receptor-1 (FEEL-1), KIAA0246, and Common lymphatic endothelial and vascular endothelial receptor-1 (CLEVER-1) [8,9,10]. Stab1 is a scavenger receptor and adhesion protein which is expressed on vascular endothelial cells, sinusoidal endothelial cells, and subsets of immunosuppressive macrophages [11,12]. STAB1 performs a multitude of functions in macrophages, including receptor-mediated endocytosis, recycling, and immune suppression [7,12]. Accumulating evidence has shown that high STAB1 expression correlates with poor prognosis in several cancers, including breast cancer, oral cancer, gastric cancer, hematological malignancies, and metastatic colorectal cancer (CRC) [12,13,14]. Studies indicate that STAB1 is associated with tumor progression by contributing to pathways involved in altered T-cell infiltration, defective antigen presentation, and resistance to immune checkpoint inhibitors [12,15]. Additionally, the inhibition of STAB1 drives immunosuppressive macrophages towards an inflammatory gene signature and promotes adaptive immune responses [7,16]. In this review, we will highlight the features of STAB1, its role in TAMs and carcinogenesis, and its potential as a targetable approach to combat existing challenges in immunotherapy.

## 2. Stabilin-1 Structure and Function

### 2.1. Overview of Stabilin-1 Structure

In 1991, STAB1 was identified as a high-molecular-mass protein and histological marker for sinusoidal endothelial cells in human spleen and named MS-1 for its antibody and protein recognition [17]. It is a multifunctional, type I membrane protein expressed by lymphatic and vascular endothelia in various organs, including liver, spleen, lymph nodes, adrenal cortex, and interstitial dendritic cells [18,19]. Also, STAB1 was found to be expressed in cutaneous T-cell lymphoma, melanoma metastasis, and chronic inflammation of the skin, such as psoriasis [8,20]. Additionally, studies have determined that STAB1 is differentially expressed on circulating monocytes and a subset of alternatively activated, type II (M2) macrophages [15,18,19,21].

STAB1 is a multifunctional class H scavenger receptor that is encoded by the *STAB1* gene located on chromosome 3p21.1 in humans [22]. The gene comprises 69 exons and encodes a protein with 2570 amino acid residues [23]. STAB1 has an intricate molecular structure with two isoforms, which could be the result of variations in post-translational modifications and alternative splicing. Western blot analyses of STAB1 confirmed the presence of two distinct bands with a tight doublet at 320 kDa and a smaller isoform at 140 kDa [24,25]. STAB1 exhibits a complex structure with a short cytoplasmic tail, a single transmembrane domain, several epidermal growth factors (EGF)-like domains, seven fasciclin domains, and a single non-functional hyaluronic acid binding X-link domain [19,26]. The complex structure of STAB1 is linked to functional properties that include immunosuppression observed during cancer. For example, STAB1 interacts with the tumor growth inhibitory factor, SPARC (secreted protein acidic and rich in cysteine) through the extracellular EGF-like domain [9]. SPARC is induced in an inflammatory environment for tissue remodeling and plays fundamental roles in development and tissue formation [27]. Consequently, the clearance of SPARC via STAB1 contributes to the regulation of ECM organization and tumor characteristics in the TME [8,10,24]. Thus, the interaction of ligands with the various domains of STAB1 contributes to the multifunctional capabilities of STAB1 and its role in tissue homeostasis as well as disease.

### 2.2. Stabilin-1: Role in Tissue Homeostasis

Because of its structural attributes, STAB1 has the capability to interact with diverse ligands to mediate endocytosis and contribute to tissue homeostasis. As a scavenger receptor, STAB1 maintains tissue homeostasis by interacting with various proteins associated with tissue inflammation and remodeling such as SPARC, stabilin-1 interacting chitinase-like protein (SI-CLP/CHID1), placental lactogen, growth differentiation factor 15 (GDF-15), transforming growth factor-β-induced protein (TGFβi), periostin (POSTN), and reelin (RELN) [16,23,28,29,30,31,32,33]. In the spleen, STAB1 expression on endothelial cells plays a critical role in controlling infiltration of B cells and CD8+ T cells into the red pulp, highlighting its essential function in immune cell trafficking and responses in the spleen [34]. In the liver, STAB1 is highly expressed on liver sinusoidal endothelial cells (LSECs) and mediates the clearance of various substances, including oxidized low-density lipoprotein from the circulation [35]. During inflammatory conditions, STAB1 can regulate the recruitment of lymphocytes, granulocytes, and monocytes from the bloodstream to the sites of injury for repair and remodeling [31,36].

For macrophages, STAB1 has been identified as a key player during tissue homeostasis. Under physiological and pathological conditions, the maintenance of tissue homeostasis necessitates the continuous turnover of biological macromolecules and associated products [23]. STAB1 is a scavenging receptor expressed on macrophages involved in the removal of harmful or excessive products [8]. For example, STAB1 can mediate the clearance and removal of bacteria [10] and specifically, lipopolysaccharide (LPS), a bacterial cell wall component [37]. Studies using *Stab1* global KO mice showed that the internalization of toxic lipid peroxidation products for degradation is impaired with the loss of STAB1-expressing macrophages [38]. In wildtype mice, the uptake of lipid peroxidation products (e.g., malondialdehyde low density lipoprotein) by STAB1+ macrophages resulted in the suppression of CCL3 secretion, a profibrogenic chemokine involved in collagen deposition in the liver [38]. Also, STAB1 has been shown to mediate the clearance of apoptotic bodies during wound healing [39]. Overall, STAB1+ macrophages maintain the tissue microenvironment by preventing oxidative damage and liver fibrosis. Thus, STAB1 exhibits multifunctional mechanisms to maintain tissue homeostasis, and the loss of STAB1 can be detrimental to tissue repair, leading to altered immune responses and disease progression, including cancer.

### 2.3. Stabilin-1: Role in Cancer Development and Metastasis

The continual rise in cancer cases and cancer-related mortality is a public health concern worldwide. Despite extensive research, the molecular mechanisms driving tumor development and metastasis remain challenging to comprehend [39,40]. The ability of tumors to evade growth suppressors, achieve replicative immortality, and avoid immune surveillance to invade secondary sites are important hallmarks of cancer progression [41]. During metastasis, tumor cells (TCs) detach from the primary site and migrate through the lymphatic and hematopoietic circulation to seed and grow in distant organs [40]. Beyond the intrinsic properties of TCs, the immune response plays a critical role in the success of cancer advancement [42]. It is known that cytotoxic and pro-inflammatory immune responses limit tumor growth, while M2 macrophages and regulatory T cells (Tregs) favor immunosuppression and TC survival [43]. The tumor-promoting processes often involve proangiogenic factors produced by M2 macrophages that stimulate tumor growth and impede effective antitumor responses [43]. Mounting evidence has shown that STAB1 is highly expressed on lymphatic vessels, M2 macrophages, and TAMs [5,42,43,44]. Thus, understanding the role of STAB1 in cancer progression is of clinical importance and can contribute to the development of new anti-cancer therapeutics.

Studies have shown high STAB1 expression in lymphatic vessels of squamous cell cancer of the head and neck, as well as breast cancer [11,45,46]. Also, STAB1 expression in lymph nodes was found to correlate with the metastatic cascade [45]. Besides promoting lymphatic metastasis, STAB1 also facilitates the adherence of cancer cells to endothelial venules in the bloodstream. This process accelerates the extravasation of cancer cells into organs, potentially resulting in hematogenous metastasis [24]. Remarkably, the contribution of STAB1 in cell adhesion and extravasation mechanisms also increases the susceptibility of tumor emboli and thrombosis development, underscoring a broad and detrimental role of STAB1 throughout disease progression [24,45]. In a clinical study on primary CRC, the involvement of lymphatic vessels was confirmed and found to be a critical prognostic factor for poor patient survival [47]. Further investigations into the role of STAB1 in immunosuppressive macrophages demonstrated that the enrichment of STAB1+ TAMs are associated with poor prognosis in breast, bladder, and oral cancers [9,11,14,48,49]. Additionally, preclinical studies using *Stab1* KO mice confirmed that the absence of STAB1+ TAMs resulted in reduced tumor burden of several cancers, including melanoma, lung adenocarcinoma, medullary mammary adenocarcinoma, and lymphoma [42,50]. Thus, the characterization of molecular mechanisms underlying immunosuppressive STAB1+ TAMs in tumor growth and metastasis may drive the development of novel anti-cancer approaches.

## 3. Macrophage Polarization and Tumor-Associated Macrophages (TAMs)

### 3.1. Macrophage Polarization

Macrophages are widely distributed in the body and are critical components of the immune system involved in both homeostasis and disease [3]. These specialized cells of the innate immune system are classified based on their differentiation into TRMs (tissue-resident macrophages) and MDMs (monocyte-derived macrophages). TRMs develop during embryogenesis and expand within the tissue through proliferation. In contrast, MDMs originate from peripheral blood monocytes that migrate into injured or infected tissues [51]. Macrophages are highly plastic and can respond to external stimuli to differentiate into M1 and M2 types [6,52]. Macrophages polarize towards the M1-like phenotype in response to LPS and cytokines (e.g., IFNγ or TNFα) [51,53]. The M1-like phenotype has been characteristically ascribed to antigen presentation, T-helper 1 (Th1) inflammatory immune activation, and the promotion of cytotoxic immune responses involving the secretion of reactive nitrogen and oxygen species [51,54]. In contrast, M2-like macrophages are activated by immune-modulatory cytokines (e.g., IL4, IL10, and IL13) and are programmed to inhibit Th1 cell activation and proliferation while promoting T helper 2 (Th2) immune responses [54]. Functionally, M2-like macrophages facilitate the clearance of apoptotic cells, promote tissue remodeling, and contribute to tumor progression [4,51,52]. Notably, the process of macrophage polarization isn’t the final stage during immune responses. Instead, it is a dynamic process that plays an important role in regulating immune responses and the signals involved in the maintenance of tissue homeostasis [53]. As such, the dysregulation of macrophage polarization can drive tumor progression since TAMs are major immune infiltrates of tumor foci that are predominantly of the immunosuppressive M2 phenotype [5,53]. Therefore, the characterization of macrophage polarization within the TME, as well as the role of TAM components including STAB1 will be advantageous in new approaches to target malignancies

### 3.2. Tumor-Associated Macrophages (TAMs)

Macrophages play a critical role in cancer-related inflammation and the regulation of tumor cell survival and expansion. However, tumor cells can exploit the dysregulation of anti-cancer immunity, programming themselves to release numerous chemotactic substances to recruit M2 immunosuppressive macrophages [47,54]. Cell recruitment involves the flux between M1/M2 phenotypes and associated cell surface markers such as STAB1 that contribute to the release of influencing signaling molecules (e.g., cytokines and chemokines) according to the makeup of the microenvironment [51].

The interplay between M1 and M2 macrophages in anti- and pro-tumor immune responses is complex [54]. Macrophages exhibit anti-tumor properties in the early stages of tumor growth and seeding [3]. However, as tumors expand, the TME is infiltrated with predominantly M2-like cells that further promote tumorigenesis and metastasis [5,54]. Mounting evidence has shown that M2 macrophages in TME are associated with poor prognoses in various cancer types, including melanoma, breast, bladder, and CRC [14,54,55]. Also, the protumor activities of M2 macrophages include the suppression of cytotoxic CD8+ T cells and the upregulation of immunosuppressive regulatory T lymphocytes [56]. During tumor expansion, M1-like TAMs are characterized by the expression of surface markers such as CD80, CD86, and CD16/32. They secrete pro-inflammatory cytokines and chemokines (e.g., TNFα, IL1β, IL6, IL12, CXCL9, CXCL10, CXCL2, CCL4, and CCL5) that are involved in tumoricidal activities [53,57,58,59,60]. In contrast, M2-like TAMs are characterized by the expression of different markers such as CD206 (Mannose receptor C-type 1, Mrc1), CD163 (scavenger receptor for hemoglobin and haptoglobin), MARCO (Macrophage receptor with collagen structure, scavenger receptor class A), HO-1(Heme oxygenase-1), and STAB1 [4,19,61,62]. Based on cytokine and signaling pathways, M2 macrophages exhibit high plasticity and are classified into four subtypes, M2a, M2b, M2c, and M2d. These subtypes are induced by distinct stimuli and are associated with specific immunoregulatory functions [63]. The polarization of M2 subtypes establishes different biological functions with activated cells releasing cytokines and chemokines (e.g., IL10, TGFα, CCL17, CCL18, and CCL22); growth factors (e.g., vascular endothelial growth factor, VEGF and platelet-derived growth factor, PDGF); as well as proteases including cathepsins and matrix metalloproteases [64,65]. Thus, M2-like macrophages play a significant role in immune regulation and the suppression of anti-tumor inflammatory signaling molecules that can contribute to tumor vascularization, growth and metastasis [4,52,54,60,64,65]. Among the M2 macrophage surface receptors, STAB1 is abundantly expressed on TAMs and implicated in protumor pathways during tumor growth and metastasis [5,56]. However, it is currently unknown if STAB1 is expressed in all M2-like cells or if it varies among the M2 subtypes. Overall, the characterization of STAB1-expressing M2 macrophages can provide key information towards the understanding of mechanisms underlying the remodeling of TME and related signaling pathways that are associated with cancer advancement.

### 3.3. Role of STAB1 in TAMs and Immune Suppression

The expression of TAMs is associated with different aspects of tumor growth and metastasis, underscoring the complicated layers of interactions between tumor cells and the surrounding environment. Further, the risk of distant metastases correlates with the density of TAMs in many malignancies. Noticeably, mounting evidence from several clinical cohorts and preclinical studies highlights the role of STAB1+ TAMs in mechanisms underlying tumor progression, thereby supporting STAB1 as a potential therapeutic target [7,9,12,14,47,48,50,66,67].

Studies on different cancers have shown that the level of STAB1-expressing TAMs correlated with disease outcomes. For instance, in a cohort of invasive breast cancer, an increased population of CD68+ and STAB1+ macrophages was observed within the intra-tumoral compartment, a region characterized by extensive stromal–parenchymal interactions. This structural composition facilitates the binding of cytokines and growth factors stimulated by the ECM to create an optimal environment for cell–stroma interactions [68]. Additionally, a clinicopathological investigation of invasive breast cancer showed a high expression of STAB1+ macrophages that correlated with poor survival [69]. Similarly, the high abundance of STAB1+ TAMs in the early stage of gastric adenocarcinoma (T1 or Tumor node metastasis (TNM) stage 1) is associated with poor survival [14]. In urothelial bladder cancer, a higher abundance of STAB1+ TAMs is linked to increased mortality following transurethral resection [55]. In a cohort study on urothelial bladder cancers, the expression of STAB1 on TAMs but not lymphatic endothelial cells correlated with poor response to neoadjuvant chemotherapy and reduced overall survival [48]. This finding opens a new avenue in translational research, highlighting STAB1 expression on TAMs as a key player in therapy resistance. Additionally, in patients with acute myeloid leukemia (AML), higher expression levels of STAB1 are significantly associated with leukocyte counts, lactate dehydrogenase levels, and poor clinical prognosis [66]. Intriguingly, the frequency of CD68+ and STAB1+ TAMs in peri- and intertumoral regions was found to be a key determinant of CRC disease stage [47]. Notably, in CRC stages I and II, a high number of CD68+ and STAB1+ TAMs in peritumoral areas was linked to increased disease-specific survival. Also, STAB1+ TAMs were found to be higher in the non-recurrence stage III. In contrast, high populations of STAB1+ TAMs in intratumor regions are associated with decreased patient survival in stage IV [47]. In a survival stage IV CRC study, further support for the relationship between STAB1+ TAMs and shorter survival from disease was observed [13]. Overall, it was determined that the location and extent of CD68+ STAB1+ TAMs could be a prognostic marker of disease stage in CRC. This accumulating evidence highlights that STAB1+ might have a role in maintaining the complexity of TME and could play a critical role in the regulation of tumor growth and metastasis (Table 1).

The expression of STAB1 in endothelial cells, lymphatic cells, and M2 macrophages in different organs and its role in tumor development is not fully characterized. In the context of melanoma tumor development, preclinical studies using *Stab1* KO mice have brought forth the following valuable information. First, studies have shown that the absence of STAB1 results in decreased tumor size in primary melanoma tumors compared to wildtype animals [42]. Second, *Stab1* KO mice exhibit reduced lymphatic metastases and decreased M2 macrophages recruitment into tumors [42]. Finally, another in vivo study using a STAB1-deficient mouse model for hepatic melanoma metastasis revealed that STAB1 modulates the deposition of ECM components, specifically POSTN and TGFβ1, which regulate immune cell infiltration and tumor growth [28]. These findings underscore the importance of STAB1 in regulating hepatic immune dynamics during melanoma metastasis.

In other studies, using conditional *Stab1* KO mice with bone marrow chimeras and cell depletion experiments in multiple solid tumors demonstrated significant impairment in tumor development with altered STAB1 expression in macrophages [50]. The deficiency of STAB1 induced the elevation of inflammatory cytokines (IL1β, IL2, IL12p70, TNFα) and chemokines (CCL3, CCL4, CCL5) in tumor-bearing mice. Also, the lack of STAB1 improved CD8+ T-cell infiltration in TME and enhanced T-cell priming in draining lymph nodes, supporting antitumor immunity [50]. Thus, STAB1 upregulation in M2 macrophages might play a critical role in the invasiveness of tumor behavior and metastasis. STAB1+ macrophages are considered pro-tumorigenic, facilitating tumor cell proliferation by suppressing immune responses in breast cancer. Specifically, STAB1 expression associates with cytotoxic CD8+ T cell inhibition and the promotion of immune regulatory T-cell (Tregs) activation [56]. The immunosuppressive function of STAB1 was supported by RNA-seq data obtained from blood samples of healthy donors and persons with tetanus vaccine and timothy grass allergy [16]. While normal blood monocytes do not express STAB1, the pro-inflammatory monocyte exhibits an upregulated expression. The downregulation of STAB1 by siRNAs (small-interfering RNA) in human monocytes resulted in the enhanced expression of several pro-inflammatory genes (e.g., ALOX5AP, PADI4, and SPP1), coupled with higher TNFα secretion and dampening of antigen-specific Th1 pro-inflammatory immune responses [16]. Subsequent investigations further reinforced the immunosuppressive role of STAB1 by demonstrating that the depletion of STAB1 in macrophages enhances the upregulation of Ifi202b (interferon-activated gene 202b) [18]. Notably, the loss of STAB1 function did not affect B cell stimulation for antibody production but instead improved the humoral immune response against tumor antigens [18]. Intriguingly, the loss of STAB1 in macrophages leads to the upregulation of M1 macrophage markers CCL3 and TNFα in the case of chronic liver injury [38]. In papillary thyroid carcinoma, enhanced M2 macrophage recruitment identified by LYVE-1 (a lymphatic vessel-specific glycoprotein), CD206, and STAB1 was observed as the disease progressed [70]. Additionally, deletion of the STAB1 gene in papillary thyroid carcinoma (PTC) by CRISPR/Cas9 decreased the CD4+/CD8+ T-cell ratio, further supporting the role of STAB1+ macrophages in immune regulation [70]. In human leukemia studies, the knockdown of STAB1 expression in AML cells resulted in enhanced apoptosis and reduced proliferation of the transformed cells. A similar finding was observed in a mouse xenograft model for STAB1 knockdown in AML cells [66]. Overall, STAB1 functions as a cargo receptor, continuously shuttling between the cell membrane and endosomal compartments to facilitate the degradation of non-self-components [8]. However, the impairment of STAB1 function may alter macrophage function with differential changes in MHC I peptide presentation and CD8 T cell activation [11,71]. As contributing mechanisms, the enhancement of mTOR signaling in *Stab1* KO mice might be involved in changes in lysosomal composition [11,50]. Also, blocking STAB1 in macrophages promotes a shift towards pro-inflammatory phenotype activation by interferon-gamma (IFNγ) signaling [7]. As reviewed in Table 2, accumulating evidence suggests that the loss of STAB1 promotes anti-tumor microenvironment in different cancers and that targeting STAB1 could be a promising approach.

## 4. Stabilin-1: Novel Approach in Immunotherapy

Over the past two decades, significant breakthroughs in cancer therapy, especially tumor immunology, have helped in understanding macrophage biology and its clinical relevance in human diseases [14,48,68,69]. An emerging immunotherapy approach to treat different forms of cancer involves methods to enhance the pro-inflammatory activity of macrophages by diminishing immunosuppressive TAMs and drug resistance [67]. Indeed, immune checkpoint inhibitors targeting programmed death (PD1) and cytotoxic T-lymphocyte-associated protein 4 (CTLA4) have been used in the treatment of several malignancies [12]. Increasing evidence implies that the TME drives drug resistance with the accumulation of myeloid cells and immunosuppressive M2 macrophages [72]. Given that current research focuses on improving immune checkpoint inhibitor efficacy, targeting STAB1 expression in TAMs could be a new strategy. Mounting evidence indicates blocking STAB1 reprograms TAMs toward an immunostimulatory (M1) phenotype with increased CD86, MHC I, MHC II and IL12p40, and IL1β, boosting CD8^+^ T-cell infiltration and antitumor activity [50,66]. Furthermore, combining anti-STAB1 antibody with anti-PD-1 therapy synergistically reduced TAMs and improved efficacy in refractory tumor models of lung and colon cancers [50]. Thus, targeting STAB1 in combination with existing immunotherapies could provide clinically relevant advancements in reducing primary tumor burden and metastases.

Notably, a humanized anti-STAB1 antibody has been evaluated in patients with various solid tumors, including immunotherapy-refractory melanoma (melanoma progression on or after anti-PD-1/CTLA-4 therapy), pancreatic-ductal adenocarcinoma, cholangiocarcinoma, hepatocellular carcinoma, ovarian carcinoma, and CRC [71]. The dysregulation of the STAB1 receptor function by anti-STAB1 antibody (FP-1305, bexmarilimab) resulted in enhanced antigen presentation and Th1 cell activity. Additionally, STAB1 inhibition resulted in the upregulation of CXCR3 in naïve CD4^+^ and CD8^+^ T-cells, the downregulation of CD206 and CD163 on CD14^HI^ cells, and the impairment of lipid metabolism in monocytes through inhibition of LXR/PXP and PPAR nuclear receptor pathways [71]. Also, downregulation of checkpoint inhibitors CTLA-4, LAG-3, PD-1, and PD-L1 on CD4^+^ T-cells occurred [71]. Further, the development of humanized anti-STAB1 IgG4 antibody is a positive result in the context of safety and tolerability with no dose-limiting toxicities [12,71]. Moreover, the MATINS (Macrophage Antibody to FP-1305 To Inhibit Immune Suppression, NCT03733990) phase I/II clinical trial with bexmarilimab supported the STAB1 blockade approach. The selected tumors evaluated were known to contain high STAB1+ TAMs and included cutaneous melanoma, gastric, hepatocellular, estrogen receptor-positive breast, and biliary tract cancers [12]. The results showed the successful activation of pro-inflammatory TAMs in patients with disease control leading to the upregulation of inflammatory cascade pathways involving T-cell receptor activation, IFN signaling genes (IFI16, IFI44, GBP1, GBP5 and CD40), immune cell attracting chemokines (CCL4L2, CCL5, CXCL9, CCL2 and CXCL16) and MHC Class I and II proteins (HLA-B, HLA-DRA and HLA-DPA1) and the activation of the adaptive immune response [12]. In preclinical in vitro studies, the inhibition of STAB1 with anti-mouse STAB1 antibody resulted in significant delays in antigen degradation and a shift towards a pro-inflammatory phenotype with increased expression of CD11c and TNFα [73].

**Figure 1:**
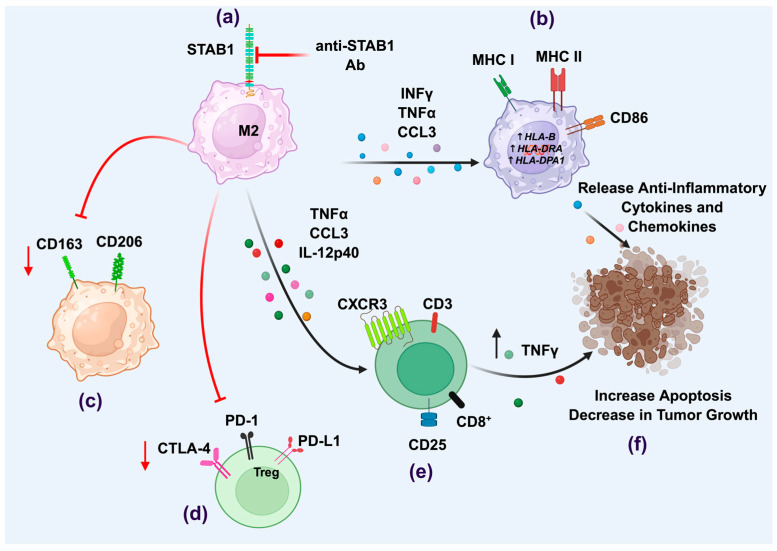
Targeting Stabilin-1 macrophage expression to enhance anti-tumor immunity. The inhibition of stabilin-1 with anti-STAB1 antibody increases the release of pro-inflammatory cytokines such as INFγ, TNFα, and CCL3, leading to a phenotypic switch of M2-type cells to anti-tumor M1 macrophages that express CD86 and antigen-presenting receptors (**a**,**b**). Inhibition of STAB1 associates with the downregulates macrophages expressing M2-type markers, CD163 and CD206 (**c**); altered Treg function (**d**); and the recruitment and stimulation of anti-tumor lymphocytes such as CD8+ T cells (**e**). Overall, STAB1 inhibition can result in decreased tumor growth and increased apoptosis of tumor cells (**f**). This figure was created with BioRender.com.

Due to the effectiveness of bexmarilimab targeting of STAB1, the utility of this therapeutic antibody is increasing for a range of cancerous disorders. Cells derived from myelodysplastic syndrome (MDS) and myeloid leukemia (AML) patient samples along with AML cell lines were treated with bexmarilimab resulted in increased higher human leukocyte antigen MHC class II (HLA-DR) expression for the promotion of antigen presentation and sensitivity to cytotoxic agents associated with cancer treatments [74]. This is in line with the results from a phase I/II trial performed in 2022–2023 in which MDS patients were treated with azacitidine alone or with azacitidine with increasing doses of bexmarilimab to assess safety and effectiveness. The combination treatment does have a manageable safety profile; thus, it may be useful for high-risk or even late-stage patients [75]. An independent study evaluating the effects of STAB1 in gastric cancer also noted that STAB1+ TAMs expressed significantly higher HLA-DR levels resulting in higher grade tumors. Blockade of STAB1 (with bexmarilimab) followed by transcriptomic analysis demonstrated that TAMs were reprogrammed to enhance production of IL1β, IFNγ, and TNFα; all pro-inflammatory and cytolytic killing of tumor cells. Tumors were also more sensitive to anti-PD-1 therapy [76]. How does bexmarilimab affect TAMs in the tumor microenvironment? Using breast cancer patient-derived explant culture (PDEC), Rannikko et al. show bexmarilimab effectiveness is TME-dependent and has differing effects on monocyte-derived macrophages versus tissue-resident macrophages. High IFN signaling and late-stage activated macrophage abundance were associated with bexmarilimab resistance, though this treatment still dampened the IFN signaling. This study shows that optimal bexmarilimab efficacy may require patient pre-treatments to obtain effective conditions in the TME for best immuno- and chemotherapeutic treatments [77]. These encouraging results in TAMs should not overshadow possible effects in other STAB1-expressing tissues. No long-term studies have analyzed the effect of an anti-STAB1 antibody on the health and function of LSECs, endothelia of lymph nodes and spleen, all tissues with high constitutive STAB1 expression levels under any physiological condition. It must be kept in mind that STAB1 is a scavenger receptor with interactions with numerous different molecules which may affect other physiological functions. Despite these cautions, targeting STAB1 represents a promising strategy for enhancing anti-tumor immunity by simultaneously modulating macrophage function and improving T-cell activation and cytotoxicity as represented in Figure 1 and outlined in Table 3.

During the writing of this manuscript, a paper from the University of Turku in collaboration with investigators at University of Birmingham announced that they had discovered a soluble form of STAB1 (sClever1) in both cell lines and patient serum. The soluble fragment consists of amino acids 26-1255 and was recombinantly generated with a C-terminal His6-tag creating a protein that is 134 kDa in size. sClever1 is slightly increased in the serum of patients with lung, skin, and breast cancers, though the source of this protein is unknown. Both cancerous cells and cells that constitutively express STAB1(ie. liver sinusoidal endothelia) may all be the source of sClever1, although the contributions by each cell population have yet to be determined. Elevated levels of sClever1 in patients correlated with resistance against anti-PD-1 therapy, though the association between survival outcomes and levels of sClever1 were not very significant due to inter-patient variations in the data set. Treatment with Bexmarilimab inhibits the release or decreases the level of sClever1 in patient serum resulting in a subset of CD8+ cells that had decreased. This is significant since sClever1 binds to the CI-IGF2R, a mannose-6-phosphate receptor, that affects the TCR activation by impairing phosphorylation at Y394 and, in turn, suppresses the function of NFAT and NFκB pathways resulting in cells that show a reduced pro-inflammatory cytokine profile [78]. The secreted version of STAB1 may be how this receptor signals to other cells via extracellular vesicles (EVs), microvesicles, or free protein in the plasma/interstitial fluid to modulate the immune profile in the tumor microenvironment. Indeed, the human STAB1 was pronounced a “dead” receptor with regards to signaling to other cellular pathways, even though it is highly endocytic and its high expression in TAMs correlated with poor outcomes in patients [9].

## 5. Conclusions

The development and metastasis of cancer cells are critical processes in disease outcomes. Cancer growth begins with cancer stem cells, fueled by cytokines, immune cells, mesenchymal cells, and stromal cells in cancer niches. TAMs are one of the key components with a high abundance in the TME that influence the plasticity and nature of the tumor mass. TAMs contribute to the complexity of cancer progression by facilitating tumor growth and creating challenges in therapeutic approaches. STAB1 is predominantly expressed in alternatively activated macrophages, which exhibit multi-functional roles in cellular trafficking, endocytosis, immune modulation, and tumor progression. The immunosuppressive role of STAB1 is evident in TAMs, where it promotes tumor progression by inhibiting cytotoxic T-cell activity and enhancing regulatory T-cell recruitment. In cancer, STAB1 has been implicated in facilitating cancer cell adhesion, transmigration, growth, and metastasis. Elevated expression of STAB1 on TAMs correlates with poor prognosis in several cancers, including breast, gastric, bladder, and CRC. Experimental models using *Stab1* KO mice resulted in a reduction in tumor growth and metastasis with enhanced antitumor immunity, underscoring its role as an immunosuppressive TAM. Mechanistically, the absence of STAB1 in macrophages enhances pro-inflammatory cytokine secretion, T-cell infiltration, and antitumor immune responses. Recent advances, such as the phase I/II MATINS clinical trial targeting STAB1 with bexmarilimab, indicate STAB1 blockade as a promising therapeutic strategy. STAB1 blockade reprograms immunosuppressive TAMs into immunostimulatory phenotypes and enhances CD8^+^ T-cell infiltration, T-cell activation, and adaptive immune responses. Thus, targeting STAB1 offers a novel strategy for cancer immunotherapy by modulating TAM-mediated immune suppression and enhancing T-cell-mediated antitumor activity. Future research can provide clinically relevant information on the mechanistic pathways of STAB1 in TAMs and its implications across various tumor types to optimize therapeutic interventions, especially in cancers resistant to conventional immunotherapies.

In summary, investigations into the role of TAMs in cancer progression have provided clinically significant information to the field of cancer immunotherapy. The identification of signaling pathways in M2 TAMs is clinically relevant, and the advent of *Stab1* KO mouse models and advanced functional and transcriptomic assessments can lead to a deeper mechanistic understanding of pro-tumorigenic TAMs and drug resistance. The study of how STAB1 activity signals the downstream regulation of pro-inflammatory genes could enhance the understanding of involved pathways. Also, a better understanding is needed of how STAB1 is involved in tumor growth, metastasis, immune modulation, and extracellular matrix remodeling. The questions regarding the regulatory role of STAB1 in modulating immune cell trafficking, tumor-associated angiogenesis, and macrophage polarization in various cancer types remain to be defined. Cutting-edge methods such as spatial transcriptomics, CRISPR-based gene editing, and single-cell proteomics may reveal complex networks of interactions and functions unique to cancer and cell type. To overcome therapeutic resistance in refractory malignancies, preclinical research may offer alternative approaches such as combinatorial treatments that target STAB1 in conjunction with immune checkpoint inhibitors or other immunotherapies. Building on the studies with bexmarilimab will be useful and provide information for STAB1 blockage in larger cohorts and a wider range of cancer types, as well as long-term implications on tumor suppression and immune reprogramming.

## Figures and Tables

**Table 1:** The expression of Stabilin-1 in tumor-associated macrophages in human cancers.

Cancer Type	Subjects (*n*)	Results	Reference
Bladder Cancer	184	Higher mortality following transurethral resection associated with STAB1^+^ TAM expression.	[55]
Bladder Cancer	68	High STAB1^+^ TAMs associated with poor survival and response to neoadjuvant therapy.	[48]
Breast Cancer	36	High expression of CD68^+^ and STAB1^+^ TAMs.	[68]
Primary Invasive Breast Cancer	148	Infiltration of STAB1^+^ TAM is associated with poor survival.	[69]
Early-stage Gastric Adenocarcinoma	371	High expression of STAB1^+^ TAMs in early-stage gastric cancer relates to poor survival rates.	[14]
Colorectal Cancer	159	CD68^+^ and STAB1^+^ TAMs in peritumoral areas associated with survival of early-stage tumors.	[47]
Colorectal Cancer	620	High CD68^+^ and STAB1^+^ TAMs correlated with poor survival.	[13]
Acute Myeloid Leukemia	87	STAB1 expression is associated with leukocyte counts, lactate dehydrogenase levels and poor survival.	[66]

**Table 2:** The role of Stabilin-1 during tumor development in rodent cancer models.

Cancer Type	Outcomes	Reference
Lung, Breast, Lymphoma, Colon Cancer	Stabilin-1 enhances tumor growth and suppresses anti-tumor immune activity.	[50]
Acute Myeloid Leukemia	Stabilin-1 suppresses anti-tumor activity.	[66]
Hepatic Melanoma Metastasis	Stabilin-1 contributes to ECM modeling, immune cell infiltration, and tumor growth.	[28]
Primary Melanoma	Reduced tumor burden with the loss of Stabilin-1.	[42]
Papillary thyroid carcinoma	Stabilin-1 supports the seeding of tumor cells.	[70]

**Table 3:** Efficacy of targeting Stabilin-1 (STAB1) expressing macrophages.

Study Model	Antibody	Findings	Reference
Rodent	Anti-STAB1 (mouse IgG1)	Enhancement of pro-inflammatory TAMs.	[50]
Rodent	Mouse IgG1 and anti-PD-1 (RMP1-14)	Reduction in immunosuppressive TAMs, and improved treatment efficacy in refractory tumor models.	[50]
Rodent	Anti-mouse STAB1 (clone 1.26)	Delayed antigen degradation and shift to pro-inflammatory macrophage phenotypes.	[73]
Clinical Trial	Humanized anti-STAB1 antibody (Bexmarilimab)	Impaired lipid metabolism in monocytes, downregulation of checkpoint inhibitors, and enhanced antigen cross-presentation.	[71]
Clinical Trial Clinical Trial Pre-Clinical Trial	Bexmarilimab Bexmarilimab and azacitidine Bexmarilimab	Upregulation of inflammatory cascade pathways and adaptive immunity. Manageable safety profile in patients with myelodysplastic syndrome (NCT05428969). Antigen presentation; increased chemotherapy sensitivity (NCT05428969).	[12] [75] [74]

## Data Availability

Data, materials, and protocols are available upon request by email to the corresponding authors due to privacy/ethical restrictions.

## Abbreviations

The following abbreviations are used in this manuscript:

AML	Acute Myeloid Leukemia
CAFs	Cancer-associated fibroblast
CLEVER-1	Common lymphatic endothelial and vascular endothelial receptor-1
CRC	Colorectal cancer
CTLA4	Cytotoxic T-lymphocyte-associated protein 4
EGF	Epithermal growth factors
EVs	Extracellular vesicles
FEEL-1	Fasciclin, EGF-like, laminin-type EGF-like and link domain-containing scavenger receptor-1
GDF-15	Growth differentiation factor 15
HO-1	Heme oxygenase-1
ICI	Immune Checkpoint Inhibitor
KO	knockout
LPS	Lipopolysaccharide
LSECs	Liver sinusoidal endothelial cells
LYVE-1	Lymphatic vessel-specific glycoprotein
MARCO	Macrophage receptor with collagen structure, scavenger receptor class A
MATINS	Macrophage Antibody to FP-1305 To Inhibit Immune Suppression
MDMs	Monocyte-derived macrophages
MDS	Myelodysplastic syndrome
Mrc1	Mannose receptor C-type 1
PDGF	Platelet-derived growth factor
PDEC	Patient-derived explant culture
POSTN	Periostin
PTC	Papillary Thyroid Carcinoma
RELN	Reelin
sClever1	Soluble form of STAB1
siRNA	small-interfering RNA
SI-CLP	Stabilin-1 interacting chitinase-like protein
SPARC	Secreted protein acidic and rich in cysteine
STAB1	Stabilin-1
TAMs	Tumor-associated macrophages
TCs	Tumor cells
Tregs	Regulatory T cells
TGFBi	Transforming growth factor-β-induced protein
Th1	T-helper-1
Th2	T-helper-2
TNM	Tumor node metastasis
TME	Tumor microenvironment
TRMs	Tissue-resident macrophages
